# Short- and long-term complications of surgical and percutaneous dilatation tracheotomies: a large single-centre retrospective cohort study

**DOI:** 10.1007/s00405-019-05394-9

**Published:** 2019-04-02

**Authors:** B. J. de Kleijn, J. Wedman, J. G. Zijlstra, F. G. Dikkers, B. F. A. M. van der Laan

**Affiliations:** 1 Department of Otorhinolaryngology-Head and Neck Surgery, University of Groningen, University Medical Center Groningen (UMCG), Hanzeplein 1, PO box 30.001, 9700 RB Groningen, The Netherlands; 2 Department of Critical Care, University of Groningen, University Medical Center Groningen, Groningen, The Netherlands; 3 Department of Otorhinolaryngology, Amsterdam UMC, University of Amsterdam, Meibergdreef 9, Amsterdam, The Netherlands

**Keywords:** Tracheotomy, Tracheostomy, Percutaneous dilatation tracheotomy, Surgical tracheotomy, Long-term complications, Short-term complications, Intraoperative complications

## Abstract

**Objectives:**

The aim of this study was to determine and compare the incidence of long- and short-term complications of percutaneous dilatation tracheotomies (PDT) and surgical tracheotomies (ST).

**Design:**

A single-centre retrospective study.

**Participants:**

305 patients undergoing a tracheotomy (PDT or ST) in the University Medical Center Groningen from 2003 to 2013 were included. Data were gathered from patient files.

**Main outcome measures:**

Short-term and long-term complications including tracheal stenosis.

**Results:**

The incidence of short- and long-term complications, including tracheal stenosis, was similar in both groups. Analysis of a small high-risk subgroup showed no difference in long-term complications.

**Conclusions:**

The rate of short- and long-term complications, including tracheal stenosis, is equal in PDT and ST. PDT is a safe alternative for ST in selected patients.

## Introduction

Tracheotomy^1^ is a surgical procedure that has been used since ancient times. It is performed for several reasons, i.e. upper airway obstruction or in case of an expected need for mechanical ventilation for more than 10–14 days [1]. It provides a safe and well-tolerated airway, providing access for pulmonary lavages, faster weaning from the ventilator and decreasing the risk of ventilator-associated pneumonia [2]. Traditionally, a surgical tracheotomy (ST) is used to perform a tracheotomy [3].

In 1969, the percutaneous dilatation tracheotomy (PDT) using the Seldinger or over-the-wire technique was developed [4]. Intensive care physicians are more familiar and comfortable with this technique and it has become a standard procedure at intensive care units (ICU) all over the world. STs are usually performed in an operation theatre (OT). The consensus is that a PDT can only be performed in stable patients without anatomical abnormalities. The PDT is, therefore, used in a selected group of patients. Performing a tracheotomy at an ICU instead of an OT implies lower cost, less persons involved and a quicker procedure [5, 6].

In the literature, there is no consensus if PDT has lower or higher complication rate [7–10]. There is little information on the long-term complications of PDT compared to ST [11–13]. A possible and serious long-term complication of tracheotomies is tracheal stenosis. When a PDT is performed there is an assumed higher risk of fracturing a tracheal ring, potentially leading to tracheal stenosis [11, 14].

This study is performed to compare the long-term complications of PDT and ST. Short-term complications are also taken into consideration.

## Materials and methods

### Inclusion

This is a retrospective study, in which a total of 305 consecutive patients undergoing tracheotomy between 2003 and 2013 were included. All included patients have had a PDT or ST in the University Medical Center Groningen (UMCG), a third-line referral hospital with 400 new patients with head and neck malignancies annually. Inclusion criteria were 18 years or older at the time of intervention and registration of technique used (PDT or ST). Variables registered were indication, anatomical abnormalities, complications, scarring, voice changes and swallowing complaints, use of anticoagulants, history of neck surgery and radiation. Patients were selected using a coded database for the ST, by doctor’s databases and a search for ‘Tracheotomy’ in electronic patient files. Radiation and/or surgery of the neck may lead to anatomical changes of the neck and are assumed to make patients unsuitable for PDT. As PDT was relatively contraindicated in patients with pre- or postoperative radiation therapy, previous neck surgery or thoracic surgery or a previous tracheotomy, these patients were excluded from the analysis and labelled as ‘high risk’. After exclusion, 189 patients were identified for analysis. The other 116 patients are included in a subgroup analysis for long-term complications.

### Definitions

Short-term complications were defined as complications within 2 weeks of surgery. They include surgical complications (false route, lacerations, bleeding), postoperative bleeding, granulation formation and infection. Long-term complications were defined as complications that appear after more than 2 weeks after surgery and can be related to tracheotomy. The 2-week time span chosen as the healing process will be mostly completed after this time. Tracheal cartilage will show effects of trauma after 2 weeks, possibly presenting in necrosis and collapse [15]. Long-term complications include tracheal stenosis, swallowing disorders, voice complaints or scarring. Swallowing disorders were described as difficulty swallowing, pain or aspiration. Voice complaints were mainly complaints of hoarseness. Swallowing and voice disorders may not be related to the tracheostomy, but to the intubation or principal problem. Therefore, only big differences between the techniques regarding these complications will be noticeable after analysis.

### Follow-up

Patients in both groups were followed until the end of the study, until death of the patient, when lost to follow-up (no records of the patients for 6 months or more) or until a new tracheotomy or a laryngectomy was performed. During regular follow-up, patients were asked for symptoms indicating long-term complications. Diagnostic procedures for the detection of complications were only performed when indicated.

### Techniques

In the UMCG, the Ciaglia Blue Rhino® (Cook medical, Limerick, Ireland) is used for performing a PDT. This is a one-step tracheal dilatator that is introduced over a guided wire and is always placed with endoscopic guidance. STs were performed using a Björk flap to prevent false routes when changing the tracheotomy tube.

### Ethical considerations

The study was approved by the Medical Ethical Committee of the University Medical Center Groningen (M13.142044). No patient consent was needed in this retrospective study.

### Analysis

Data were gathered using Microsoft Access 2010 and statistical analysis was performed using IBM SPSS Statistics 22. Several methods were used to analyse the differences between the two techniques. The Chi-squared test was used for ordinal data and independent samples *T* test was used for normally distributed continuous data. The Kolmogorov–Smirnov test of normality was used to determine non-parametric distribution. A Mann–Whitney *U* test was used to compare the two groups for non-parametric data.

## Results

### Baseline characteristics

As shown in Table 1, 52.9% of included patients underwent ST. Almost all patients in the IC unit have prophylactic anticoagulants administered to prevent thrombosis. In Table 1, ‘anticoagulant use’, therefore, is limited to therapeutic anticoagulant use (acenocoumarol or heparin).

**Table 1:** Registered baseline characteristics of 189 study subjects who underwent either PDT or ST

PDT	47.1% (*n* = 89)			
ST	52.9% (*n* = 100)			
	PDT	ST	*p* value*	Total group
Male	*n* = 56 (62.9%)	*n* = 64 (64.0%)	0.878	63.5%
Age during surgery (means in years)	59.8	56.0	0.07	57.8
Anticoagulant use	*n* = 1 (1.1%)	*n* = 3 (3.0%)	0.371	2.1%
Obesity	*n* = 8 (8.9%)	*n* = 9 (9.0%)	0.998	8.9%

*PDT* percutaneous dilatation tracheotomy, *ST* surgical tracheotomy

*A Mann–Whitney *U* test was used to compare the age during surgery (non-parametric data).The Chi-squared test was used for ordinal data

### Indication

As shown in Table 2, the indication for performing the tracheostomies is different for both techniques. The ST was more often used in patients with ENT tumours. PDT is used in selected patients of the ICU when the expected need for mechanical ventilation is longer than 14 days.

**Table 2:** Indication for tracheotomy in a cohort of 189 subsequent patients

Indication	PDT (*n* = 89)	ST (*n* = 100)	*p* value*
Oncology	*n* = 1 (1.1%)	*n* = 27 (27.0%)	< 0.001
Postoperative	*n* = 9 (10.1%)	*n* = 1 (1.0%)	0.005
Neurology	*n* = 5 (5.6%)	*n* = 26 (26.0%)	< 0.001
Benign obstruction	*n* = 0 (0%)	*n* = 12 (12.0%)	0.001
Vascular	*n* = 5 (5.6%)	*n* = 6 (6.0%)	0.911
Pulmonary	*n* = 37 (41.6%)	*n* = 12 (12.0%)	< 0.001
Swallowing	*n* = 1 (1.1%)	*n* = 0 (0%)	0.288
Others	*n* = 27 (30.3%)	*n* = 16 (16.0%)	0.019
Not registered	*n* = 4 (4.5%)	*n* = 0 (0.0%)	0.032

*PDT* percutaneous dilatation tracheotomy, *ST* surgical tracheotomy

*The Chi-squared test was used for ordinal data

### Short-term complications

No statistically significantly differences in short-term complications were registered in either group (Table 3). Perioperative complications consisted of tracheal laceration or airway obstruction during surgery. In one patient in the ‘high-risk’ group, it was necessary to convert the PDT to an ST. The conversion was performed because of tracheal laceration with positioning of the tracheotomy tube in the oesophagus. One other patient in the ‘high-risk’ group needed surgical intervention days after the PDT because of narrow tracheal opening rendering switching of the tracheotomy tube difficult. Neither reintervention had long-term sequelae.

**Table 3:** Short-term complications in a cohort of 189 subsequent patients

Complication	PDT (*n* = 89)	ST (*n* = 100)	*p* value*
Perioperative complications	*n* = 3 (3.4%)	*n* = 6 (6.0%)	0.397
Operative major bleeding	*n* = 0 (0.0%)	*n* = 1 (1.0%)	0.344
Postoperative granulation	*n* = 3 (3.4%)	*n* = 9 (9.0%)	0.113
Postoperative infection	*n* = 0 (0.0%)	*n* = 2 (2.0%)	0.180

Major bleeding is defined as bleeding during the procedure that requires ligation of vessels or surgical intervention. Normal haemostasis during surgery is not taken into account

*PDT* percutaneous dilatation tracheotomy, *ST* surgical tracheotomy

*The Chi-squared test was used for ordinal data

### Long-term complications

Only patients with a follow-up of more than 2 weeks were included for the analysis of long-term complications, leaving 87 PDT and 84 ST patients. Patient that had complaints of swallowing or voice before removal of the airway cannula were excluded as the cannula influences swallowing and voice quality. All long-term complications registered were comparable between the two tracheotomy techniques (Table 4).

**Table 4:** Long-term complications in a subgroup of the study population (*n* = 171 of total population) that have a follow-up of 2 weeks or more, and a ‘high-risk’ subgroup (*n* = 107) also with a follow-up of 2 weeks or more

Complication	PDT (*n* = 87)	ST (*n* = 84)	*p* value*
Long-term complications			
Tracheal stenosis	*n* = 3 (3.4%)	*n* = 4 (4.8%)	0.665
Swallowing disorders	*n* = 2 (2.3%)	*n* = 1 (1.2%)	0.581
Voice complaints	*n* = 1 (1.1%)	*n* = 0 (0.0%)	0.324
Scarring	*n* = 1 (1.1%)	*n* = 9 (10.7%)	0.014

Complication	PDT (*n* = 14)	ST (*n* = 93)	*p* value*
Long-term complications in ‘high-risk’ subgroup			
Tracheal stenosis	*n* = 0 (0%)	*n* = 4 (4.3%)	0.429
Swallowing	*n* = 0 (0%)	*n* = 4 (4.3%)	0.429
Voice complaints	*n* = 0 (0%)	*n* = 3 (3.2%)	0.495
Scarring	*n* = 2 (14.3%)	*n* = 6 (6.5%)	0.299

*PDT* percutaneous dilatation tracheotomy, *ST* surgical tracheotomy

*The Chi-squared test was used for ordinal data

Tracheal stenosis was registered in three patients (3.4%) in the PDT group and four (4.8%) in the ST group (*p* = 0.665, Chi-squared test). Routine examination of the trachea did not take place. In the ST group, one patient had a subclinical (i.e. had no complaints) tracheal stenosis. This stenosis was discovered by laryngeal endoscopy for other reasons. The proportion of patients that certainly was not having a tracheal stenosis, confirmed by endoscopic laryngeal examination, was 4.5% for PDT and 3.0% for ST. In the majority of the patients, tracheal stenosis was not objectified by direct observation, or the findings were not reported. The number of subclinical stenosis can, therefore, not be assessed.

### High-risk patients

The above-analysed group consisted of all patients who underwent a PDT or ST without pre- or postoperative radiation therapy, previous neck surgery or thoracic surgery or a previous tracheotomy. Patients who underwent pre- or postoperative radiation therapy, previous neck surgery or thoracic surgery or a previous tracheotomy can be determined as high risk. Using these criteria, 107 high-risk patients were identified. Sub-analysis is shown for high-risk patients (Table 4). The patients that had a PDT are small (*n* = 14) in this group as the risk factors are a relative contraindication to perform a PDT. No statistically significantly differences were found.

### Follow-up

**Fig. 1:**
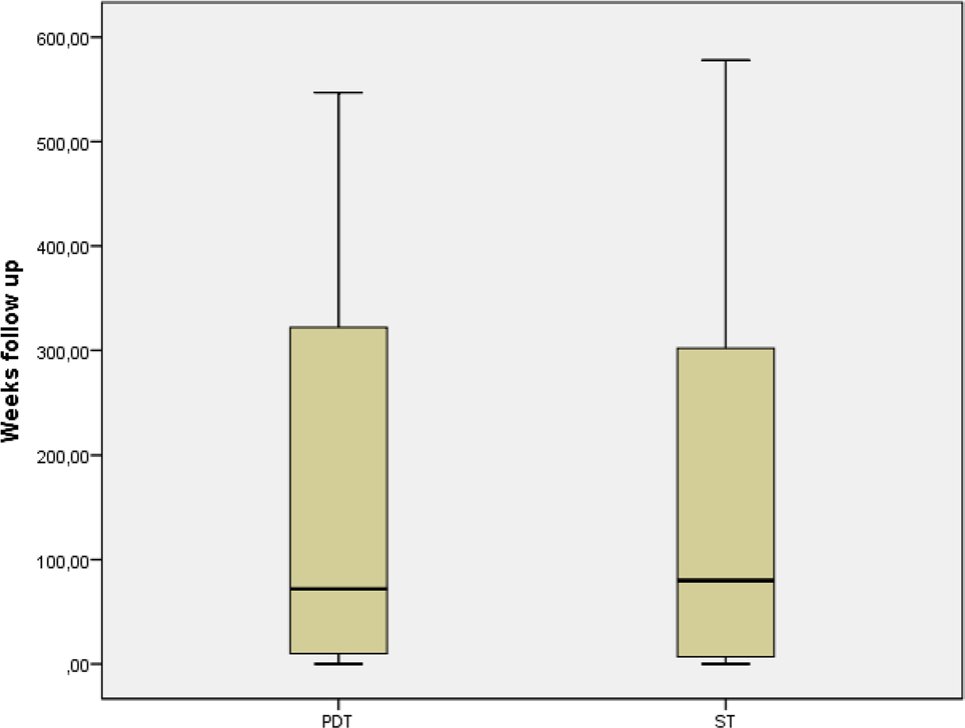
Box plot for follow-up in weeks. PDT (percutaneous dilatation tracheotomy): mean 99.23. ST (surgical tracheotomy): mean 91.24. Distribution is the same across the groups (*p* = 0.316, Mann–Whitney *U* test for non-parametric distributed continuous data)

Follow-up rates were comparable (Fig. 1). There are many reasons for ending follow-up. The main reason is death of the patient. The reasons for ending follow-up were comparable (Table 5). The amount of patients that died within follow-up of this study is comparable in both groups (*p* = 0.188, Chi-squared test). Median time to death after tracheotomy was 6 months for PDT patients and 5 months for ST patients, with a similar distribution (Mann–Whitney *U* test, *p* = 0.278).

**Table 5:** Reasons for ending follow-up in a cohort of 189 subsequent patients

Reason ending follow-up	PDT (*n* = 89)	ST (*n* = 100)	*p* value*
Deceased	*n* = 45 (50.6%)	*n* = 41 (41.0%)	0.188
Lost to follow-p	*n* = 20 (22.5%)	*n* = 28 (28.0%)	0.383
New tracheotomy	*n* = 3 (3.4%)	*n* = 5 (5.0%)	0.579
Laryngectomy	*n* = 0 (0%)	*n* = 6 (6.0%)	0.019
End of study	*n* = 21 (23.6%)	*n* = 207 (20.0%)	0.549

*PDT* percutaneous dilatation tracheotomy, *ST* surgical tracheotomy

*The Chi-squared test was used for ordinal data

## Discussion

As this was a retrospective study, no randomisation between the two techniques was performed. The indication for performing the tracheotomy is different in both groups (Table 2). This is mainly because a PDT is performed on stable patients in the ICU when the expected need for mechanical ventilation is longer than 14 days. A ST is performed in ENT patients and in patients that are not on the ICU or have a high-risk profile. To limit the bias caused by the difference in indication to perform the tracheotomy, high-risk patients were excluded from analysis. Still, the indication differs between the groups. A ST is statistically significantly more often performed in case of oncology, neurologic problems and benign airway obstruction. A PDT is statistically significantly more often performed for pulmonary reasons or postoperative. As tumours may change anatomy and can cause a change in routine, a bias is introduced, leading to a more favourable outcome for PDT patients. This bias can only be prevented by performing a randomized study.

This retrospective study allowed for analysis of baseline, operative, short-term and long-term characteristics. There was no significant difference in perioperative complications. Conversion to a surgical procedure during PDT or surgical intervention after PDT is rare, with 2% in our total series. This is comparable with Voelker et al. [16]. A limitation of this retrospective study is that the information about perioperative complications of the ST was primarily found in surgical reports and no structured reports were made for PDT. There is probably a substantial discrepancy in registration, especially of minor complications.

There is a lack of consensus in the literature regarding short-term complications in PDT compared to ST. Several studies show more short-term complications in ST patients [9, 17, 18]. Oliver et al. found more early complications in PDT compared to ST [19]. Other studies do not show any differences [6, 7, 20–22]. The meta-analysis by Higgins et al. illustrated no clear difference, but a trend toward fewer short-term complications in PDT [20]. In our study, short-term complications do not differ statistically significantly. The lack of consensus in the literature could be explained by the more accurate registration during and after ST compared to PDT. Also, a ST is more often performed in high-risk patients with specific comorbidities and tumours in the neck region. In our study, these patients were excluded from the analysis.

An important outcome measure in our analyses concerned the presence of tracheal stenosis after tracheotomy. Tracheal stenosis can lead to shortness of breath. Depending on the severity, patients either have no discomfort, or have shortness of breath during exercise or even when resting. Many patients will not notice a small degree of stenosis, depending on their physical exercise capacity. It is to be expected that patients report symptoms of clinical stenosis during follow-up visits. Subclinical stenosis may be missed if patients are not examined for tracheal stenosis. As this was a retrospective study, no screening tests were performed to detect tracheal stenosis. Most subclinical stenoses were, therefore, not detected. It is to be expected that a proportion of patients have subclinical tracheal stenosis.

Only two studies have compared the long-term complications between PDT and ST [19, 23]. Both studies used pooled data and showed that PDT and ST have a comparable number of long-term complications. Our article is the only original article using single-centre data to compare long-term complications. In our study, we found 3.4% tracheal stenosis in patients after PDT and 4.8% after ST (not statistically significant), most of them with clinical symptoms. Low rates of clinical tracheal stenosis after PDT have been described in the literature [11–13]. Young et al., performing magnetic resonance imaging of 50 patients that underwent a PDT ≥ 3 months before, found a stenosis rate of 10%, none of them showing clinical symptoms [24]. A subgroup analysis of high-risk patients was performed for long-term complications. No statistically significant differences were found, but the PDT group is small (*n* = 14). PDT is less often performed in this high-risk group as pre- or postoperative radiation and previous surgery causes scarring, fibrosis, atrophy and changes the anatomy of the neck. This can make a PDT more difficult to perform and facilitates long-term complications such as tracheal stenosis. PDT is, therefore, mainly used in patients with a low-risk profile. The use of PDT has been extended to higher risk patients in recent years, there are reports showing the safety of using PDT in patients after thoracic organ transplant procedures [25]. We believe the use of PDT will be extended to higher risk patients in coming years.

## Strengths and limitations

This study was performed in a large cohort. All patients above 18 years old in a large medical centre were included and sub-analysis was performed for high-risk patients. It is a retrospective study, so registration bias is expected. Also, as all ST patients were traceable from operation logs, none will have been overlooked. PDT patients were gathered using a patient file search as no records were held. Some PDT patients may, therefore, have been overlooked. Not all patients were examined postoperatively for subclinical tracheal stenosis. Therefore, subclinical stenosis could be underrepresented in this study.

## Conclusion

This study shows PDT as a safe alternative to ST in selected patients. The rate of short-term and long-term complications including tracheal stenosis is equal in PDT and ST. We believe the use of PDT will be extended to higher risk patients in coming years.

## Footnotes

1 The procedure is called tracheostomy or tracheotomy in the literature. We reserve the term ‘tracheostomy’ for the airway stoma of laryngectomized patients.
